# Body computed tomography in sepsis: predictors of CT findings and patient outcomes in a retrospective medical ICU cohort study

**DOI:** 10.1007/s10140-022-02083-9

**Published:** 2022-08-04

**Authors:** Julian Pohlan, Denis Witham, Lara Farkic, Melina Anhamm, Alexandra Schnorr, Gloria Muench, Karim Breiling, Robert Ahlborn, Enrico Herz, Kerstin Rubarth, Damaris Praeger, Marc Dewey

**Affiliations:** 1 Department of Radiology, Charité – Universitätsmedizin Berlin, corporate member of Freie Universität Berlin, Humboldt-Universität Zu Berlin, and Berlin Institute of Health, Campus Charité Mitte, Berlin, Germany; 2 Berlin Institute of Health at Charité –Universitätsmedizin Berlin, Charitéplatz 1, 10117 Berlin, Germany; 3 Institute of Biometry and Clinical Epidemiology, Charité – Universitätsmedizin Berlin, corporate member of Freie Universität Berlin and Humboldt-Universität Zu Berlin, Charitéplatz 1, 10117 Berlin, Germany; 4 Department of Cardiology With Intensive Care, Charité – Universitätsmedizin Berlin, Campus Charité Mitte, Berlin, Germany; 5 Department of Information Technology, Charité – Universitätsmedizin Berlin, Campus Charité Mitte, Berlin, Germany

**Keywords:** Sepsis, Computed tomography, Intensive care, Infectious focus

## Abstract

**Background:**

Sepsis is a life-threatening condition that requires immediate focus identification and control. However, international sepsis guidelines do not provide information on imaging choice.

**Purpose:**

To identify predictors of CT findings and patient outcomes in a population of septic patients from a medical ICU.

**Material and methods:**

A full-text search in the radiological information system (RIS) retrieved 227 body CT examinations conducted to identify infectious sources in 2018. CT reports were categorized according to identified foci and their diagnostic certainty. Diagnostic accuracy of CT was compared to microbiological results. Clinical and laboratory information was gathered. Statistical analysis was performed using nonparametric tests and logistic regression analysis.

**Results:**

CT revealed more positive infectious foci 52.4% (*n* = 191/227) than microbiological tests 39.3% (*n* = 79/201). There were no significant differences between focus-positive CT scans with regard to positive microbiological testing (*p* = 0.32). Sequential organ failure assessment (SOFA) scores were slightly but nonsignificantly higher in patients with a focus-positive CT, odds ratio (OR) = 0.999 (95% CI 0.997–1.001) with *p* = 0.52. Among C-reactive protein (CRP), procalcitonin (PCT), and leukocytes, in focus-positive versus focus-negative CT scans, CRP showed a minor but statistically significant elevation in the group with focus-positive CT scans (OR = 1.004, 95% CI = 1.000–1.007, *p* = 0.04). No significant association was found for PCT (OR = 1.007, 95% CI = 0.991–1.023; *p* = 0.40) or leukocytes (OR = 1.003, 95% CI = 0.970–1.038; *p* = 0.85). In 33.5% (*n* = 76/227) of cases, the CT findings had at least one therapeutic consequence. In 81.6% (*n* = 62/76), the CT findings resulted in one consequence, in 14.5% (*n* = 11/76) in two consequences, and in 3.9% (*n* = 3/76) in three consequences. There was no significant association between focus-positive CT scans and mortality (*p* = 0.81).

**Conclusion:**

In this population of septic patients in medical intensive care, microbiological analysis complemented CT findings. Both clinical and laboratory parameters were not predictive of CT findings. While therapeutic consequences of CT findings in this study population underline the role of CT for decision making in septic patients, CT findings do not predict patient outcomes in this retrospective analysis.

**Supplementary Information:**

The online version contains supplementary material available at 10.1007/s10140-022-02083-9.

## Introduction

Sepsis requires diagnostic workup and initiation of treatment to eliminate the underlying causal infection[1]. In untreated patients, sequential organ failure due to mostly bacterial, but also fungal, parasitic, or viral infection, may lead to septic shock and death [2, 3]. While advocating fast source control, international sepsis guidelines do not provide any recommendations on the choice of imaging modality in septic patients [4].

Treatment of septic patients in an intensive care unit (ICU) is performed for both community-acquired sepsis and sepsis due to hospital-acquired infection [5]. Among the known risk factors for sepsis, immunosuppression and age have been emphasized [6–8]. Septic encephalopathy or ventilation may hamper adequate clinical assessment [9, 10], whereas body computed tomography (CT) is a versatile tool that may detect a wide range of possible underlying causes. Although many authors have described the role of CT in sepsis [11–13], a comprehensive analysis of patient outcomes in relation to CT findings has not been published before.

We previously provided a detailed analysis of the diagnostic yield of CT in septic ICU patients and investigated the role of CT in patients from the emergency department compared with patients treated in regular wards [14–16] Whereas common septic sources in surgical patients are known to include surgical site infections, imaging findings in patients from a medical ICU remain scarcely studied.

This study aims at identifying predictors of CT findings and patient outcomes in a population of septic patients from a medical ICU.

## Material and methods

### Study design

In a retrospective study, we analyzed CT reports of patients examined in a large university medical center. The study design and data from this patient population and analysis have been published previously [14]. All adult inpatients 18 years or older referred for CT examinations from a medical intensive care unit (ICU) over a 12-month period from Jan. 1 through Dec. 31, 2018, were considered for enrollment, and we included all body CT examinations conducted to identify infectious sources. We excluded referrals from general wards and the emergency department as well as outpatients. Also, the analysis did not include patients from surgical or neurological ICU so to avoid a bias by surgical site infections. Body CT examinations include at least the chest and abdomen and may optionally be supplemented by any additional region/s. Ten CT examinations were excluded after full review of the reports: five cases due to no infection suspected/no focus search, one case as no body CT scan was performed (only chest CT), three cases due to trauma scan (after resuscitation), one case due to staging request. Two hundred twenty-seven reports were finally included in this retrospective analysis [14]. Whereas the previously published manuscript focused on diagnostic accuracy data of CT in sepsis, all analyses provided in the “Results” section have not been reported before.

### Ethics

The local ethics committee approved the study. Analysis was performed in accordance with the Declaration of Helsinki. Informed consent was waived.

### Identification of cases

We performed a full-text search of the radiological information system (RIS; CentricityTM RIS-I 6 2018, General Electric Company) to identify all relevant patients with sepsis who underwent body CT [14]. The full-text search was designed to be broad, thus not all search terms needed to be present. The authors assessed all cases identified by the full-text search individually and excluded cases according to previously defined criteria. Diagnosis of sepsis was confirmed using sepsis-related organ failure assessment (SOFA) scores, as documented. The cases identified included patients referred for body CT with a clinically undefined focus and/or inconclusive prior ancillary diagnostic findings (e.g., x-ray, ultrasound, microbiology). In 58.6% of the identified cases (*n* = 133/227), the referral included further requests, i.e., tumor, ischemia, pulmonary artery embolism, and bleeding. Forty-five patients with two (19.4%; *n* = 32/165), three (5.5%; *n* = 9/165), and four (2.4%; *n* = 4/165) CT scans during the study period were enrolled several times, resulting in a total of 227 CT examinations performed in 165 patients. One patient was examined during two different hospitalization periods and was therefore counted as two patients. CT examinations performed only for follow-up were excluded to focus on patients with only clinical and/or laboratory signs of infection at the time of the initial scan. Reports are referred to as cases in the following.

### Computed tomography

All CT scans were performed in a large university medical center on four different Aquilion scanners (Canon Medical Systems, CA, USA) with 64 to 320 detector rows. Multiplanar reconstructions were generated according to institutional standards at 1-mm and 5-mm slice thickness in axial, sagittal, and coronal reformations. An iodine-based contrast agent (Iopromide, Ultravist 370, Bayer, Leverkusen, Germany) was administered intravenously (weight-adjusted dosage). As previously reported, in 99.1% (*n* = 225/227) of CT scans, intravenous contrast agent was used; 0.1% of CT examinations (*n* = 2/227) were performed as unenhanced scans due to contraindications to intravenous contrast agent [14].

In general, delays of 60 to 90 s after injection were used for scanning the chest and abdomen. When pulmonary embolism had to be ruled out, the chest was imaged as a triggered series, followed by a scan of the abdomen with the above delay. CT of the head for focus search was performed with a delay of 40 to 180 s (after an unenhanced series). CT of the neck was performed with a split bolus and a delay of 70 s. No adverse events were reported in this population.

### Data collection

Clinical data, including routine patient demographic data, were extracted from the hospital information system (Table 1). CT request forms and patient notes were searched to retrieve immunocompromising factors, including known medical conditions and medications. All microbiological tests at the time of the CT examinations were extracted (plus/minus 1 day). Contaminations such as *Staphylococcus epidermidis* in one of two blood culture samples or *Candida* in bronchoalveolar lavage were excluded. Therapeutic consequences were researched in patient notes and defined as patient management associated with the CT result, performed within 3 days after the CT scan, i.e., antibiotic therapy change and infectious source control such as surgery or drainage. The following clinical parameters were assessed: length of hospital stays, CT-related interventions such as percutaneous drainage or surgery up to 3 days after CT examinations, and in-hospital mortality.

**Table 1:** Basic characteristics of the study

Variable		Number	Percentage
Patients		165	
Sex	Male	98	59.4%
	Female	67	40.6%
Immunosuppression	Yes	68	41.2%
	No	93	56.4%
	Not documented	4	2.4%
Further requests	Yes	133	58.6%
	No	94	41.4%
Mean duration of hospitalization in days		46.5 (*39.9*)	
Discharge	Home	44	26.7%
	For further treatment (rehabilitation center = 15, other hospital = 13, geriatrics = 10, nursing home = 1, palliative care = 2, other = 1)	42	25.5%
	Information missing	5	3.0%
Referral to regular ward	Yes	94	57.0%
	No	71	43.0%
In-hospital mortality	Died	74	44.8%
	Survived	91	55.2%

Population and morbidity/mortality outcomes; results in absolute numbers and percentages or averages (standard deviation). Part of the demographic analysis has been published elsewhere (14). Discharge home refers to patients’ discharge following hospitalization with intermittent referral to regular wards

*SD*, standard deviation

### Data analysis

A detailed description of the analysis has been published previously [14]. Briefly, the CT examination reports were separately analyzed and categorized by two readers, one medical student, and one radiologist. Readers were blinded to the final diagnosis and therapy or any therapeutic consequences of the CT findings while analyzing and categorizing CT reports. Inconclusive cases refer to reports that received two different evaluations. For all inconclusive cases, imaging data were re-read. In all cases, consent was ultimately reached among the medical student, radiologist, and clinician.

CT findings were classified both by localization of the infectious source according to body region and by diagnostic confidence regarding focus identification. Focus identification was assessed on a four-point scale: no focus of infection is found (0), a possible focus can be identified [1], the scan provides a likely infectious focus [2], or the scan shows a definite focus [3]. The grading typically reflected the radiologists’ level of confidence based on the extent of the organ involvement based on previously validated criteria. If more than one focus was identified, each was graded individually according to diagnostic confidence.

### Statistical analysis

Collected data were captured in Excel (Microsoft, Office 365 MSO, Microsoft, Redmond, WA, USA, Version 1908, 2016). Data protection was guaranteed. Categorical data are presented as numbers and percentages per group, while biomarker levels are expressed as medians and interquartile ranges. The final discharge diagnosis from the discharge note was used as the reference standard for CT findings.

Proportions of cases in groups and subgroups were analyzed using contingency tables. The chi-squared test was used to compare differences in frequencies, including analysis of in-hospital mortality. Laboratory findings were analyzed with binary logistic regression. Due to the high correlation of the parameters, these were analyzed in separate univariable using logistic regression models. Post hoc testing was done with Mann–Whitney *U* test corrected using Bonferroni correction.

Due to the exploratory nature of our study, *p* values are to be interpreted as exploratory, not confirmatory. Statistical analysis was performed using SPSS (IBM Statistics software, IBM Corporation, Version 25, 2017). A *p* value < 0.05 was considered statistically significant for all tests. Two-tailed tests were performed, unless mentioned otherwise.

## Results

### Basic characteristics

This analysis included 227 CT examinations in 165 patients; further details have been published elsewhere [14]. Patients were hospitalized for a mean of 46.5 days (standard deviation (SD) 39.9). Of patients, 44.8% (*n* = 74/165) died during their hospitalization (Table 1). A focus was identified in 84.1% of CT scans (*n* = 191/227), while no focus was detected in 15.9% of CTs (*n* = 36/227).

### Predictors of CT findings

Patients with immunocompromising conditions were analyzed according to whether CT findings were positive or negative: there was no significant difference regarding CT focus identification between patients with (43.6%, *n* = 99/227) and without immunosuppression (53.3%, *n* = 121/227): *p* = 0.88, median = 2.0 (interquartile range (IQR) = 1–3). SOFA scores were slightly but nonsignificantly higher in patients with a focus-positive CT, odds ratio (OR) = 0.999 (95% CI 0.997–1.001) with p = 0.52. We then analyzed laboratory parameters indicative of infection, i.e., C-reactive protein (CRP), procalcitonin (PCT), and leukocytes, in focus-positive versus focus-negative CT scans: CRP showed a minor but statistically significant elevation in the group with focus-positive CT scans (OR = 1.004, 95% CI = 1.000–1.007, *p* = 0.04). No significant association was found for PCT (OR = 1.007, 95% CI = 0.991–1.023; *p* = 0.40) or leukocytes (OR = 1.003, 95% CI = 0.970–1.038; *p* = 0.85).

### Microbiological testing

**Fig. 1:**
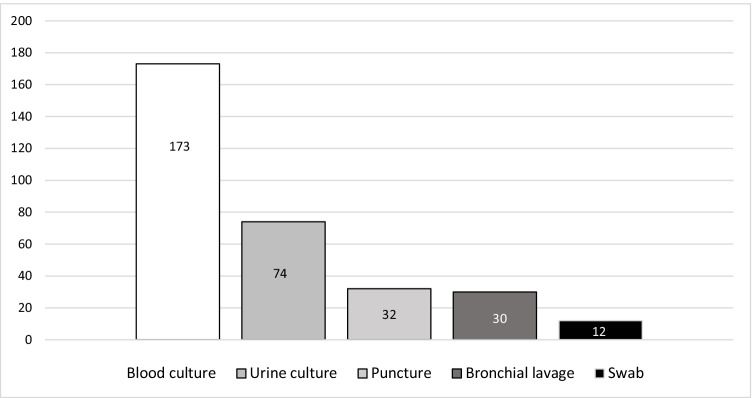
Types of microbiological assessment (*n* = 321). The bar chart depicts in detail what types of microbiological assessment were performed. The majority of microbiological tests performed around the time of CT examination were blood cultures, accounting for 53.9% (*n* = 173/321). Other tests included urine culture, 23.1% (*n* = 74/321); puncture, 10.0% (*n* = 32/321); bronchial lavage, 9.4% (*n* = 30/321); and swabs, 3.7% (*n* = 12/321)

**Fig. 2:**
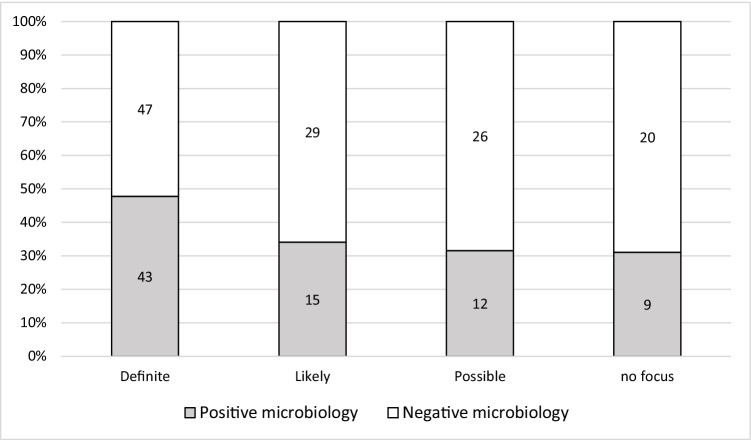
Diagnostic confidence level for focus-positive CT and results of microbiological analysis (*n* = 201). The bar chart indicates whether the microbiological analysis yielded a positive result in different levels of confidence in CT performed for focus search, i.e., the microbiological yield dependent on the imaging results. A definite focus on CT was associated with positive microbiology testing in 47.8% (*n* = 43/90); a likely focus on CT with positive microbiology in 34.1% (*n* = 15/44); and a possible focus with positive microbiology in 31.6% (*n* = 12/38). There was no focus on CT but positive microbiology in 31.0% (*n* = 9/29). A higher share of positive microbiological testing can be appreciated for definite foci on CT. Still, no significant association between different levels of confidence and associated microbiology findings was observed; chi-squared test with *p* = 0.32

Complete analysis of all microbiological testing performed at the time of the CT examination identified 201 microbiological tests in 227 cases. The majority of microbiological tests were blood cultures (Fig. 1). In 44.8% (90/201) of cases, microbiology showed growth of gram-positive/gram-negative bacteria or fungi, while 55.2% (*n* = 111/201) of the microbiological analyses remained sterile. After excluding contaminations, 39.3% (*n* = 79/201) positive microbiological tests were counted as infections. A positive microbiology test result was noted in 29.6% of tests (*n* = 95/321), corresponding to at least one positive test result in 34.8% of CT examinations (*n* = 79/227). Of all identified pathogens, 43.2% (*n* = 41/95) were gram-positive, 42.1% (*n* = 40/95) were gram-negative, and 14.7% (*n* = 14/95) fungal infections. There were no significant differences in microbiological findings between focus-positive CTs at different confidence levels versus negative CTs (*p* = 0.32). The number of positive microbiological tests was considerably lower than the number of positive CT scans (Fig. 2).

### Consequences of CT examinations

In 33.5% (*n* = 76/227) of cases, the CT findings had at least one therapeutic consequence. In 81.6% (*n* = 62/76), the CT findings resulted in one consequence, in 14.5% (*n* = 11/76) in two consequences, and in 3.9% (*n* = 3/76) in three consequences. A total of 93 consequences were found to be related to addressing the infectious focus. Therapeutic consequences followed in 36.1% (*n* = 69/191) of focus-positive CT scans versus 19.4% of focus-negative CT scans (*n* = 7/36). The most common consequence was a change in the patients’ anti-infective regimen (suppl. Table 1). The second most common consequence was surgical or interventional treatment.

### Patient outcomes

For morbidity and mortality outcomes, length of hospital stays and in-hospital mortality were analyzed in conjunction with CT findings: focus-positive CTs were not significantly associated with the duration of hospitalization with *p* = 0.13. The length of hospital stay was at a mean of 49.5 days (SD 55.0) in the focus-positive and 46.4 days (SD 50.5) in the focus-negative CT group. Also, there was no significant association between focus-positive CT findings and in-hospital mortality with *p* = 0.81: i.e., patients with focus-positive CT died in 46.1% (*n* = 88/191), patients with focus-positive CT survived in 53.9% (*n* = 103/191).

## Discussion

**Fig. 3:**
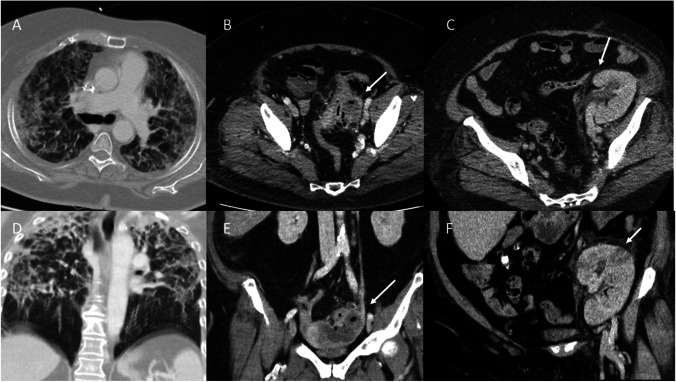
Imaging examples of patients with sepsis who received diagnostic CT. Patient 1 was a 62-year-old female with infiltrates confined to the upper lobes of both lungs consistent with a diagnosis of pneumonia (**a** axial 5-mm reconstruction with lung kernel, **d** coronal plane); this patient died during hospitalization. Patient 2 was a 61-year-old female with a fluid-collection in contact both with the colon sigmoideum and the left adnexa; the imaging differentials included both an abscess due to perforated diverticulitis and a tubo-ovarian abscess (**b** axial 5 mm with soft tissue kernel, **e** coronal 5 mm), with the latter being confirmed by surgery. Patient 3 was a 69-year-old male with peripheral perfusion deficits and perirenal fluid (arrows) in a kidney transplant (**c** axial 5 mm with soft tissue kernel, **f** coronal 5 mm)

In this population, CT was more often positive for an infectious focus than microbiological testing, confirming the sensitivity of CT for focus detection. CT findings in medical ICU patients with sepsis cannot be accurately predicted from the clinical presentation including immunosuppression or laboratory parameters. In a significant subset of patients, CT findings resulted in a modification of the anti-infective regimen and surgical or interventional treatment. CT findings did not allow the prediction of patient outcomes such as mortality (Fig. 3).

To our knowledge, there are no studies comparing the diagnostic yield of microbiology and CT in ICU patients with sepsis. We previously analyzed CT findings compared with microbiology in patients with suspected sepsis in the emergency department [15]. The results of the current study suggest that CT and microbiological testing may have complementary roles as patients with a focus-negative CT may have a positive culture and vice versa [17].

To our knowledge, patient outcomes in relation to CT findings have not been reported in the literature before.

The retrospective design of this study does not allow interpretation of associations as causal links between the factors analyzed. This study was designed to generate hypotheses from the explorative analysis. The number of chest foci was high in our analysis, though a complete analysis of risk factors for pneumonia was not performed. Ventilation as one factor known to predispose patients to nosocomial pneumonia was not assessed. This study aimed at accounting for the heterogeneity of sepsis, and a more detailed analysis of specific foci was beyond the scope of this manuscript. The analysis of mortality is probably biased by therapeutic interventions aiming the focus detected by CT. Due to the heterogeneity of the data, it was not possible to account for this factor appropriately; a prospective study design might prove advantageous. Based on the results from this study and previous analyses, our data point to a predictive role of quantifying reader’s diagnostic confidence and thereby supporting structured reporting in imaging report. Even if it may be helpful for clinicians to consider while establishing a differential diagnosis, these data currently only represent single center data.

In conclusion, CT was more sensitive in focus detection than microbiological analysis, but the two diagnostic methods provide complementary information. Both clinical and laboratory parameters were not predictive of CT findings. In many patients of this cohort, treatment decisions were based on CT findings. CT findings seem to be insufficient predictors of patient outcomes.

## Supplementary Information

Below is the link to the electronic supplementary material.

Supplementary file1 (PDF 214 KB)

## References

[1] Liu VX, Fielding-Singh V, Greene JD (2017). The timing of early antibiotics and hospital mortality in sepsis. Am J Respir Crit Care Med.

[2] Lagunes L, Encina B, Ramirez-Estrada S (2016). Current understanding in source control management in septic shock patients: a review. Ann Transl Med.

[3] Gotts JE, Matthay MA. 2016 Sepsis: pathophysiology and clinical management. Bmj ;353. 10.1136/bmj.i1585 27217054

[4] Singer M, Deutschman CS, Seymour CW (2016). The Third International Consensus Definitions for Sepsis and Septic Shock (Sepsis-3). JAMA.

[5] Vincent J-L, Sakr Y, Sprung CL (2006). Sepsis in European intensive care units: results of the SOAP study*. Crit Care Med.

[6] Poutsiaka DD, Davidson LE, Kahn KL (2009). Risk factors for death after sepsis in patients immunosuppressed before the onset of sepsis. Scand J Infect Dis.

[7] Martin GS, Mannino DM, Moss M (2006). The effect of age on the development and outcome of adult sepsis. Crit Care Med.

[8] Papadimitriou-Olivgeris M, Aretha D, Zotou A (2016). The role of obesity in sepsis outcome among critically ill patients: a retrospective cohort analysis. Biomed Res Int.

[9] Eidelman LA, Putterman D, Putterman C (1996). The spectrum of septic encephalopathy: definitions, etiologies, and mortalities. JAMA.

[10] Karlsson S, Varpula M, Ruokonen E (2007). Incidence, treatment, and outcome of severe sepsis in ICU-treated adults in Finland: the Finnsepsis study. Intensive Care Med.

[11] Schleder S, Luerken L, Dendl LM (2017). Impact of multidetector computed tomography on the diagnosis and treatment of patients with systemic inflammatory response syndrome or sepsis. Eur Radiol.

[12] Barkhausen J, Stöblen F, Dominguez-Fernandez E (2016). Impact of CT in patients with sepsis of unknown origin. Acta Radiol.

[13] Just KS, Defosse JM, Grensemann J (2015). Computed tomography for the identification of a potential infectious source in critically ill surgical patients. J Crit Care.

[14] Pohlan J, Witham D, Opper Hernando MI, Muench G, Anhamm M, Schnorr A, Farkic L, Breiling K, Ahlborn R, Rubarth K, Praeger D, Dewey M (2022). Relevance of CT for the detection of septic foci: diagnostic performance in a retrospective cohort of medical intensive care patients. Clin Radiol.

[15] Pohlan J, Witham D, Muench G, Kwon HJ, Zimmermann E, Böhm M, Praeger D, Dewey M (2021 Jan) Computed tomography for detection of septic foci: retrospective analysis of patients presenting to the emergency department. Clin Imaging 69:223–227. 10.1016/j.clinimag.2020.09.004 10.1016/j.clinimag.2020.09.004 32971451

[16] Pohlan J, Hernando MIO, Hogrebe A, Witham D, Muench G, Kwon HJ, Goehler F, Marek A, Praeger D, Dewey M (2020) The role of body computed tomography in hospitalized patients with obscure infection: Retrospective consecutive cohort study. Eur J Radiol 132 10.1016/j.ejrad.2020.109325 33027726

[17] Ahvenjärvi L, Laurila J, Jartti A (2008). Multi-detector computedtomography in critically ill patients. Acta Anaesthesiol Scand.

