# Supplementary material for: Body computed tomography in sepsis: predictors of CT findings and patient outcomes in a retrospective medical ICU cohort study
Source: Emerg Radiol. 2022 Aug 4;29(6):979–85. doi: 10.1007/s10140-022-02083-9 (PMC9643202; doi:10.1007/s10140-022-02083-9)
Supplement: Supplementary file 1 — Supplementary file1 (PDF 214 KB) [file 10140_2022_2083_MOESM1_ESM.pdf]

Supplementary Table 1: Therapeutic and diagnostic consequences of CT findings. Anti-infective regimen, intervention, further diagnostic tests are provided. Information were retrieved from patient files.

**Anti-infectives**	n=58/93 (62.4%)
Antibiotics (initiation=33, escalation=9, change=5, continued=2, discontinued=1, change & initiation=1)	51
Antimycotics (initiation=5, change=2)	7
**Interventions**	n=32/93 (34.4%)
Surgery (cholecystectomy, intestinal resection, percutaneous nephrostomy, laparoscopy, lavage)	14
Drainage (CT-guided insertion, intensified flushing)	9
Puncture (e.g., fluid aspiration, biopsy)	5
Catheter removal	3
Surgical abscess drainage	1
**Further diagnostic tests**	n=3/93 (3.2%)
Microbiological assessment of urine	1
Upper endoscopy	1
Bronchoscopy	1
No consequence	75
Not documented	76

Supplementary table 2: Radiation dose exposure per CT scan and cumulated per patient.

	CT tube radiation output in mGy*cm	Estimated effective dose in mSv	CT-identified foci	Total number
Median per CT scan (IQR)	1101.9 (488.5-1795.8)	11.0 (7.3-17.1)	1.0 (1.0-2.0)	227
Median per patient during hospital stay (IQR)	1346.2 (588.6-2055.5)	14.1 (7.9-22.1)	1.0 (1.0-2.0)	165

~~Radiation exposure~~

Although no radiation dose restraints exist for patients requiring diagnostic CT, patients who underwent more than one body CT examination in our study population already exceeded the limit of 20 mSv set for occupational exposure to ionizing radiation (18,19). This should remind radiologists and clinicians alike to reduce exposure to a necessary minimum. On the other hand, the high rate of focus-positive CT scans especially in patients who underwent repeat CT imaging justifies the extra ionizing radiation exposure in this patient population. Patients underwent a median of 1 (IQR=1-2) CT examinations for focus search during hospitalization. A higher number of CT scans resulted in a higher median dose exposure ($p<0.001$). Exposure was estimated to be 11.0 mSv (SD 7.3-17.1) per CT scan, resulting in a total of 14.1 mSv (SD 7.9-22.1) per patient ~~(table 2)~~. A larger number of CT scans per patient was associated with a higher diagnostic yield,

i.e., the number of foci identified by CT, Spearman's rank correlation with $r=0.53$, $p<0.001$ (fig. 3). Medians (IQR) are provided. CT-identified infectious foci are provided. The Kruskal-Wallis test was applied for analyzing radiation dose. All CT examinations were requested for septic patients treated in a medical intensive care unit.

IQR - interquartile range
